# Supplementary material for: Incorporation of tetracarboxylate ions into octacalcium phosphate for the development of next-generation biofriendly materials
Source: Commun Chem. 2021 Jan 11;4:4. doi: 10.1038/s42004-020-00443-5 (PMC9814588; doi:10.1038/s42004-020-00443-5)
Supplement: Supplementary file 2 — Supplementary Information [file 42004_2020_443_MOESM2_ESM.pdf]

Supplementary Information

Incorporation of tetracarboxylate ions into octacalcium phosphate for the development of next-generation biofriendly materials

Taishi Yokoi[1,2*], Tomoyo Goto[3], Mitsuo Hara[4], Tohru Sekino[3], Takahiro Seki[4], Masanobu Kamitakahara[5], Chikara Ohtsuki[6], Satoshi Kitaoka[2], Seiji Takahashi[2] and Masakazu Kawashita[1]

[1] Institute of Biomaterials and Bioengineering, Tokyo Medical and Dental University (TMDU), 2-3-10 Kanda-Surugadai, Chiyoda-ku, Tokyo 101-0062, Japan

[2] Materials Research and Development Laboratory, Japan Fine Ceramics Center, 2-4-1 Mutsuno, Atsuta-ku, Nagoya 456-8587, Japan

[3] The Institute of Scientific and Industrial Research, Osaka University, 8-1 Mihogaoka, Ibaraki 567-0047, Japan

[4] Department of Molecular and Macromolecular Chemistry, Graduate School of Engineering, Nagoya University, Furo-cho, Chikusa-ku, Nagoya, 464-8603, Japan

[5] Graduate School of Environmental Studies, Tohoku University, 6-6-20 Aoba, Aramaki, Aoba-ku, Sendai 980-8579, Japan

[6] Department of Materials Chemistry, Graduate School of Engineering, Nagoya University, Furo-cho, Chikusa-ku, Nagoya 464-8603, Japan

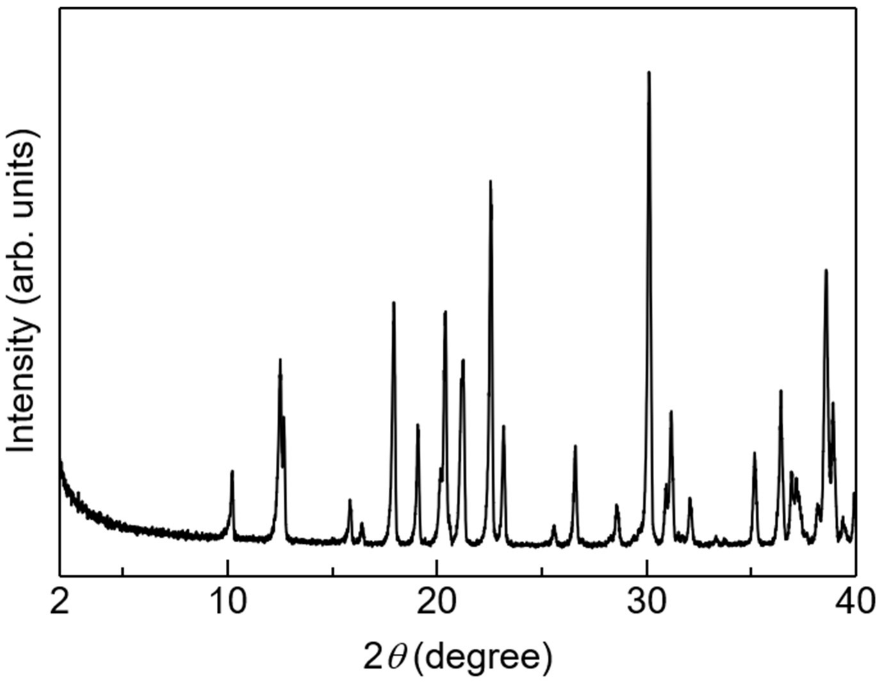

Supplementary Figure 1: Powder XRD pattern of calcium pyromellitate. Calcium pyromellitate was synthesised by mixing the following solutions at 60.5 °C: 5 cm^3 of 1 $mol{\cdot}dm^{-3}$ $CaCl_2$ solution and 200 $mol{\cdot}m^{-3}$ of acetate buffer solution containing 5 $mol{\cdot}m^{-3}$ pyromellitic acid (pH=5.5).

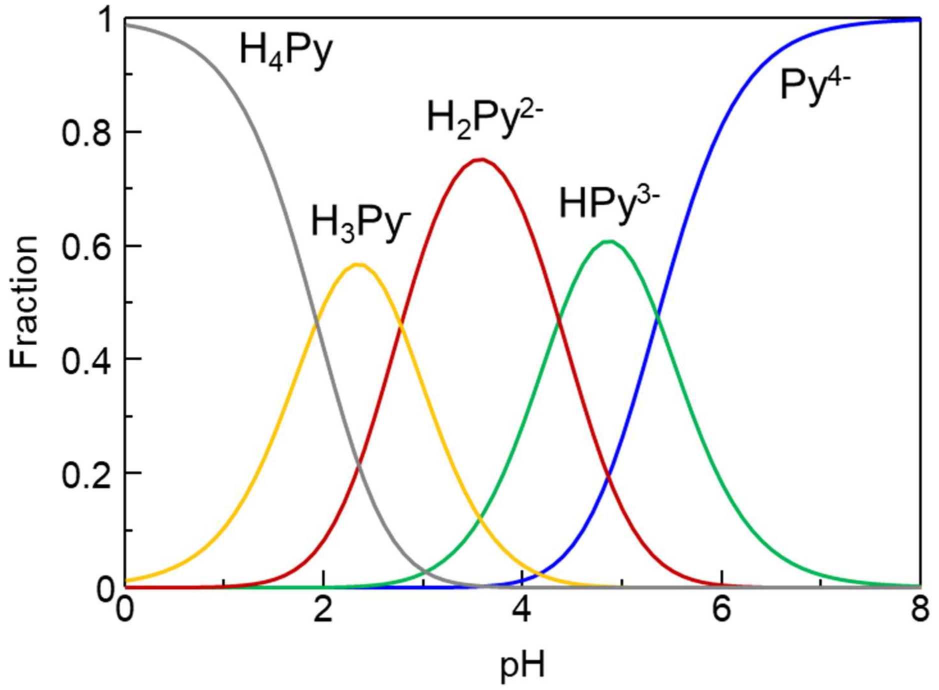

Supplementary Figure 2: Dissociation states of pyromellitate ions with respect to the pH. The dissociation states of pyromellitic acid were calculated based on the following dissociation constants: pK_{a1}=1.92, pK_{a2}=2.77, pK_{a3}=4.36, and pK_{a4}=5.35 [Ref. 39].

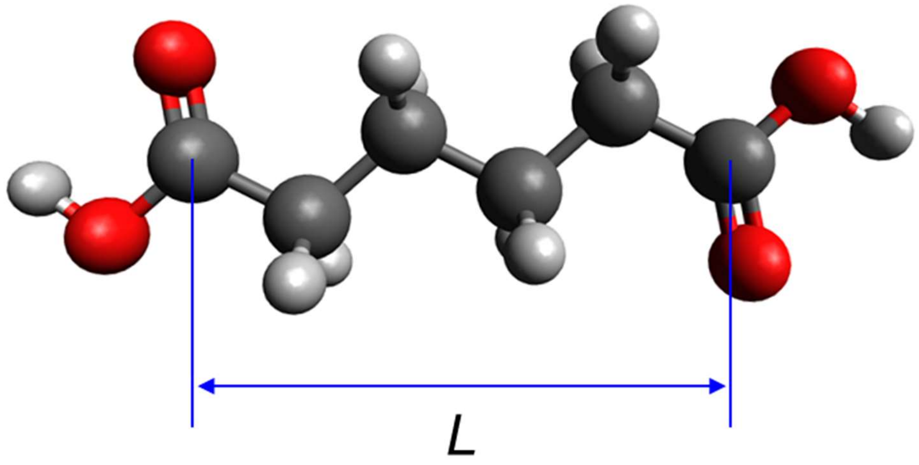

Supplementary Figure 3: Schematic illustration showing the definition of *L*. *L* is the distance between the carbon atoms of two carboxy groups of a carboxylic acid (in this case adipic acid).
